# Antimicrobial Action of Essential Oil of *Tagetes minuta*: Role of the Bacterial Membrane in the Mechanism of Action

**DOI:** 10.3390/antibiotics14070632

**Published:** 2025-06-21

**Authors:** Anahí Bordón, Sergio A. Rodríguez, Douglas Siqueira de Almeida Chaves, Andrea C. Cutró, Axel Hollmann

**Affiliations:** 1 Laboratorio de Compuestos Bioactivos, Centro de Investigación en Biofísica Aplicada y Alimentos (CIBAAL), Universidad Nacional de Santiago del Estero, Consejo Nacional de Investigaciones Científicas y Técnicas (CONICET), RN 9 km 1125, Santiago del Estero 4206, Argentina; ani.bordon.1996@gmail.com; 2 Instituto de Ciencias Químicas, Facultad de Agronomía y Agroindustrias, Universidad Nacional de Santiago del Estero, CONICET, RN 9 km 1125, Santiago del Estero 4206, Argentina; 3 Pharmaceutical Science Department, Health and Biological Science Institute, Federal Rural University of Rio de Janeiro, BR 465, km 7, Seropédica 23897-000, Rio de Janeiro, Brazil; chavesdsa@ufrrj.br; 4 Facultad de Ciencias Médicas, Universidad Nacional de Santiago del Estero, Calle Reforma del 18 N° 1234, Santiago del Estero 4200, Argentina; 5 Laboratorio de Microbiología Molecular, Universidad Nacional de Quilmes. Roque Sáenz Peña 352, Bernal 1876, Argentina

**Keywords:** antibacterial, volatile compounds, membrane damage, essential oil, natural products

## Abstract

Background: The rise in multidrug-resistant bacteria has intensified the search for new antibiotics, drawing attention to essential oils (EOs) for their antimicrobial properties. For this reason, this study focuses on the antimicrobial action of the EO obtained from *Tagetes minuta* and its impact on bacterial membranes. Methods: The EO was chemically characterized by chromatography–mass spectrometry, and its antimicrobial activity and its effects on surface and bacterial membrane were assessed by using Zeta potential, membrane transition temperature (Tm) determination; and fluorescence spectroscopy with Laurdan and Di-8 ANEPPS. Results: Twenty-seven compounds could be identified, with (E)-Tagetone, (Z)-Ocimenone, and β-pinene as the most abundant. Afterward, the EO was tested against *Escherichia coli* (MIC and MBC = 17 mg/mL) and *Staphylococcus aureus* (MIC = 8.5 mg/mL; MBC > 17 mg/mL), showing antimicrobial action in both bacteria, being more effective against *E. coli*. Mechanistic studies revealed that the EO interacts with bacterial membranes, increasing the Zeta potential by more than 9 mV and enhancing membrane permeability up to 90%. These effects were further confirmed using model lipid membranes, where the EO induced significant changes in membrane properties, including a reduction in dipole potential and transition temperature, suggesting that some EO components could be inserted into the lipid bilayer, disrupting membrane integrity. Conclusions: The EO from *T. minuta* demonstrates efficient antimicrobial activity by compromising bacterial membrane structure, highlighting its potential as a natural antimicrobial agent.

## 1. Introduction

The increasing prevalence of multidrug resistance against traditional antibiotics among pathogenic bacteria has become a health problem and heightened the demand for new antibiotic sources [1]. In this context, plant-derived metabolites have attracted growing interest because of their antimicrobial activity. They comprise families of different bioactive molecules, known as secondary metabolites, which have been shown to act on multiple bacterial targets, leading to growth inhibition or cell death [2]. In addition, the attention to plant-derived drugs has also been reinforced by the recent global trend away from synthetic medicine and towards herbal therapy, often referred to as a growing preference for plant-based therapies [3]. 

Among natural plant metabolites, essential oils (EOs), a mixture of different compounds from plant metabolism that can be obtained from different tissues and organs of plants via physicochemical and mechanical processes [4], are of the utmost importance [5]. In general, EOs are often liquids at normal temperatures, with colors ranging from pale yellow to emerald green and blue to dark brownish, depending on the plant type producing them [6]. In this sense, there has been a rising interest in the antibacterial action of EOs, with numerous studies demonstrating their antibacterial properties. However, specific mechanisms for impairing bacterial growth of EOs remain largely unknown [7]. 

*Tagetes minuta* L., commonly known in Argentina as “chinchilla”, is an annual aromatic herb from the family Asteraceae and the genus Tagetes, which is one of the most common plant taxonomical groups, with more than 1000 genera and over 23,000 species [8]. The plant is native to South America and is present in Argentina, Chile, Bolivia, and Peru. This plant has traditionally been used to treat several ailments, such as the flu or cough, as well as to heal wounds and allergies [9,10]. Different authors have reported that the EO of *T. minuta* contributes to various pharmacological activities, including antimicrobial [11,12,13,14,15]. These properties and possible benefits of *T. minuta* derivatives have attracted the attention of researchers, pharmacologists, and herbal medicine practitioners for different applications in healthcare, pharmacology, and natural product development [16]. However, despite the several works that support the *in vitro* evidence of the antimicrobial effectiveness of EO of *T. minuta* against bacteria, more research is needed to fully investigate the potential of this plant. Understanding the mechanism of action of its pharmacological effects and discovering molecular targets is crucial to achieving their full potential within the medical, cosmetics, and aromatherapy sectors. In addition, the food manufacturing areas continue to pursue natural preservatives and other agents that may substitute the synthetic compounds currently employed. The fact that many EOs are listed as generally recognized as safe (GRAS) reinforces their potential in the food industry [16,17,18].

In this sense, this work aimed to characterize the antimicrobial action of the EO obtained from the aerial parts of *T. minuta* from Santiago del Estero (Northwest Argentina), with special emphasis on the role of the bacterial membrane in the mechanism of the antibacterial action of this EO. *Escherichia coli* and *Staphylococcus aureus* were used as models of Gram-negative and Gram-positive bacteria, respectively. To deeply unravel the effect of this EO on the membrane structure, model lipid membranes were also employed. 

## 2. Results and Discussion

### 2.1. EO Characterization

The extraction yield of *T. minuta* was 0.69% (*w*/*w*). Twenty-seven compounds were identified and quantified in the EO (Table 1), with (*E*)-Tagetone (16.6%), (*Z*)-Ocimenone (12.57%), β-pinene (bicyclic monoterpene, 12.34%), α-Terpinolene (10.75%), Limonene (7.93%), and Thymol (6.12%) being the most abundant, which constitute 66.3% of the whole EO. 

Previously, the chemical profile of EO from *T. minuta* obtained from different locations has been studied and compared, showing significant variances that have been related to a variety of human and environmental factors [8]. In this regard, several studies reported different compositions, but in most of them, Dihydrotagetone, (*E*)-Tagetone, (Z)-Tagetone, Limonene, (*Z*)-Ocimenone, (*E*)-Ocimenone, and β-Ocimene are present but with different proportions [8,13,14,15,19] in agreement with our results. For instance, a study that compared the EOs obtained from aerial parts of *T. minuta* located in Egypt, South Africa, and the UK found that they had similar constituents, differed in proportion, and had Dihydrotagetone as the most abundant component for the oils from the UK (34.3 and 54.1%). On the other hand, the EOs from Egypt and South Africa were characterized by a high content of *cis*-β-ocimene (32.0 and 50.9%, respectively) [14]. Other studies have explored the chemical profiles of *T. minuta* grown in three states in India at varying altitudes. The major compounds were *Z*-β-ocimene (56.34–39.32%), Dihydrotagetone (28.07–7.66%), *E*-Ocimenone (25.06–0.00%), and *Z*-Tagetone (14.46–5.29%). A significant altitudinal impact was observed with higher *Z*-β-Ocimene concentrations from high altitudes, while the opposite was found in the case of Dihydrotagetone [13].

### 2.2. Antibacterial Activity 

The antibacterial activity of *T. minuta* EO was tested on *S. aureus* and *E. coli*, using the microdilution test, and the minimum inhibitory and bactericidal concentrations were determined. It was observed that the EO has shown inhibitory and bactericidal action (i.e., MIC and MBC) on *E. coli* at the same concentration. In contrast, only an inhibition value was obtained for *S. aureus* as no MBC was recorded at the maximal concentration tested (Table 2). In this regard, *T. minuta* EOs collected from India demonstrated greater effectiveness against Gram-positive bacteria than Gram-negative bacteria [13]. Similarly, EOs are typically more effective against Gram-positive bacteria than Gram-negative bacteria. The difference in effect between bacteria may be due to variances in their cell envelopes. The outer membrane and the presence of lipopolysaccharides in that membrane of Gram- negative bacteria helps to be more “impermeable” to components of EOs, making it less susceptible [20]. However, considering now the MBC data, the EO under study showed a higher lethal activity over *E. coli*; these data depart from the typical behavior of EOs. In this regard, some investigations have also shown that some EO components, which have high lipophilicity and a low molecular weight, can pass through membranes and exert antimicrobial effects [21]. In the same way, it was suggested that some hydrophobic substances could pass the outer membrane via porin channels [22]. Moreover, other phenomena beside the structural difference in the bacterial envelope can also be implied.

Regarding previous studies with *T. minuta* EO, Abdoul-Latif et al. (2022) have reported that EO, obtained from Djibouti (North Africa), showed inhibitory activity against *E. coli* and *S. aureus*. In this case, 13 compounds were identified as main components, including Dihydrotagetone (20.8%), Artemisia (17.9%), (Z)-Tagetone (12.4%), (-)-Spathulenol (11.0%), and Estragole (9.5%), which constitute 71.6% of the EO [9]. EOs obtained from the UK, Egypt, and South Africa, showed antimicrobial action, mainly against Gram-positive bacteria, being greatest in the EO from the UK [14]. In that study, the MIC values ranged from 25 to 100 µg/mL, which are lower than those obtained in the present work [14]. This could be attributed to differences in the chemical composition of *T. Minuta* EO. The EO used in this study contains lower concentrations of Tagetone, Ocimenone, and Dihydrotagetone, which are compounds with antibacterial activity. These results reinforce the idea that differences in the EO composition affect its antimicrobial potency. Such alterations may be attributed to adaptation to specific habitats, combined with plant age and genetic variety [15]. Nonetheless, the MICs reported here are similar to, or even lower than, those reported for other EOs such as *Origanum vulgare, Allium sativum, Cinnamon*, and *Lime* EO, among others [23,24]. It should be pointed out that MIC values of vegetable extract are typically higher than antibiotics [25]; however, EOs could show benefits over conventional antibiotics, especially regarding resistance. In this regard, EO obtained from leaves of *T. minuta* has also exhibited high antibacterial action against methicillin-resistant *S. aureus* [19]. 

**Figure 1:**
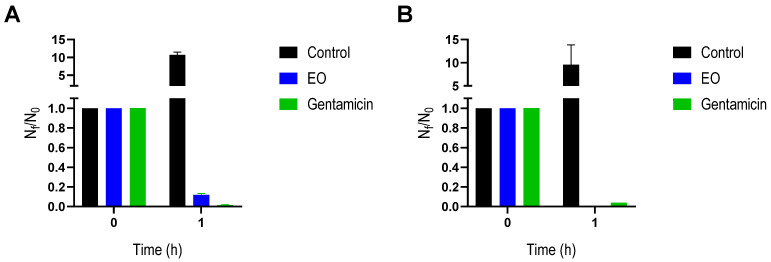
Bacterial viability after 1h of incubation with *Tagetes minuta* EO. (**A**) *Staphylococcus aureus* and (**B**) *Escherichia coli* in the presence of EO at MIC and its absence (control) are represented as blue and black bars, respectively. Gentamicin, also at MIC, was also included as a control. N_f_ and N_o_ are the final and initial counts of CFU, respectively. The data presented are an average of three separate measurements, and the error bars show the standard deviation of the averaged results.

The MIC and MBC values represent binary endpoints of measurement (growth/no growth) [26]. Consequently, for a better evaluation of the behavior of bacterial species studied in the presence of EO, viability studies were carried out by incubating both bacteria with EO at MIC for 1 h. From the results obtained for control experiments (with DMSO) after 1 h of incubation, there was remarkable bacterial growth, as expected. However, the presence of *T. minuta* EO was able to decrease the viability of both bacteria, representing a 90% and 100% reduction for *S. aureus* and *E. coli*, respectively (Figure 1). These data show the lethal effect of this EO on both bacteria, comparable with the commercial antibiotic gentamicin, being complete for *E. coli*, consistent with the MBC data obtained above. These results reinforce the potential of this EO as an antimicrobial, as it is widely recognized that rapid kinetics of bacterial death may hinder the emergence of resistance [27,28].

### 2.3. Interaction of EO with Bacterial Envelope

**Figure 2:**
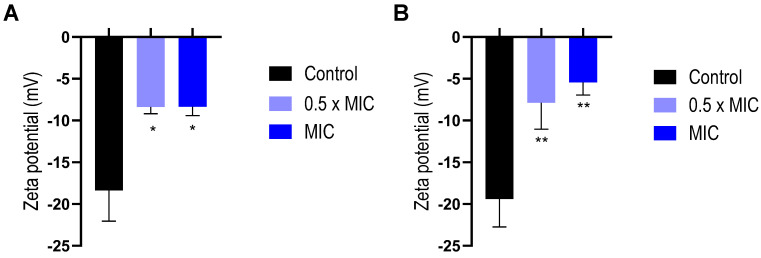
Bacterial Zeta potential as a function of *Tagetes minuta* EO concentrations. (**A**) *Staphylococcus aureus* and (**B**) *Escherichia coli*. Control represents the bacteria without EO. The data presented are an average of three separate measurements, and the error bars show the standard deviation of the averaged results. **, *p* < 0.05. *, *p* < 0.01. One-way ANOVA followed by Dunnett’s test for multiple comparisons vs. control.

The interaction of antibacterial compounds with bacterial surfaces could be considered the first step in the general mechanisms for their biological action, which could be accompanied by changes in cell surface properties, such as the Zeta potential [29]. For this reason, the determinations of the Zeta potential at different EO concentrations after 1 h of incubation in both bacterial models were performed. Zeta potential values obtained without EO were −18.4 mV and −19.4 mV for *S. aureus* and *E. coli*, respectively, which is in good agreement with previous publications [30,31,32]. On the other hand, 17 mg/mL of EO in PBS showed a Zeta potential value of −1.8 ± 0.6 mV. Significant changes in Zeta potential values were recorded by the action of the EO on the cell surface, even at the lower concentration tested (0.5 × MIC). For both bacteria, EO interactions with the bacterial surface resulted in less negative Zeta potential values compared to bacterial controls (Figure 2). 

Those changes observed in the Zeta potential could be ascribed to the interaction of some components of the EO with the bacterial envelope. Similar behaviors on the bacteria’s surface were described for other EOs with antibacterial activity, such as the EO obtained from *Schinus areira* [33] or *Lavandula angustifolia* [34]. 

### 2.4. Effect of EO on Bacterial Membrane

**Figure 3:**
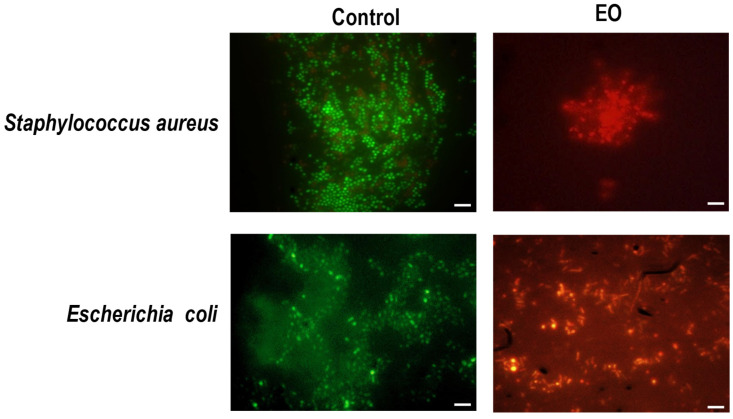
Fluorescence microscopy images of planktonic cells of *Staphylococcus aureus* and *Escherichia coli* stained using the probes SYTO-9 and PI after incubation in the presence of EO and DMSO. Scale bar: 5 μm.

In order to obtain a better description of the EO’s effect on the bacterial membrane and its relationship with the antibiotic activity, both bacteria used were incubated with the EO at MICs (i.e., 8.5 mg/mL and 17.0 mg/mL for *S. aureus* and *E. coli*, respectively) for 24 h and then dyed with SYTO-9 and PI. These fluorescence probes allow the differential staining of cells with intact membranes (green) from those in which membrane integrity is compromised (red). The images obtained indicated that this EO increased the membrane permeability in both types of bacteria, as was evidenced by the red fluorescence observed in all the treated cells in comparison with the controls, where the cells presented green fluorescence after the incubation time (Figure 3). 

Moreover, the relative membrane damage was estimated as the ratio of the green (510 nm) and red (620 nm) emission fluorescence intensities of the stained bacterial cells incubated with this EO at MIC for 1 h. It was observed that the cells with membrane damage in *S. aureus* represented 38.1% ± 2.2, whereas in *E. coli*, this proportion rose to 90.5% ± 5.4 (Figure S1). These results are consistent with the fact that *T. minuta* EO exhibits a differential antibacterial action on *E. coli* in comparison to *S. aureus*, where after 1 h of incubation, the bactericidal action on *S. aureus* is less pronounced than in *E. coli* (Figure 2). As was pointed out above, these differences could be related to a difference in the bacterial structures and the EO component interaction with the bacterial surface and membrane.

Overall, these results indicate that the EO can injure the membranes of both bacteria tested. This point is in line with previous studies for other EOs, which pointed out that the bacterial membrane is one of the key targets for exerting its antibacterial action [35,36]. In addition, some EO components, such as Limonene [37] and Thymol [38], also showed the ability to increase bacterial membrane permeability. Furthermore, these findings confirm a previous hypothesis for the antibacterial mechanism of *T. minuta* EO, which attributed the interaction of this EO with phospholipids in cell membranes, leading to increased permeability and leakage of cytoplasmic contents, to the bactericidal action [17]. 

### 2.5. Effect of EO on Mimetic Lipid Membrane

Considering that changes in membrane permeability are generally related to modifications of lipid bilayer properties such as fluidity, thickness, phospholipid mobility, and membrane polarity, the effect of *T. minuta* EO on mimetic liposomes of DMPC: DMPG (5:1) was evaluated. 

**Figure 4:**
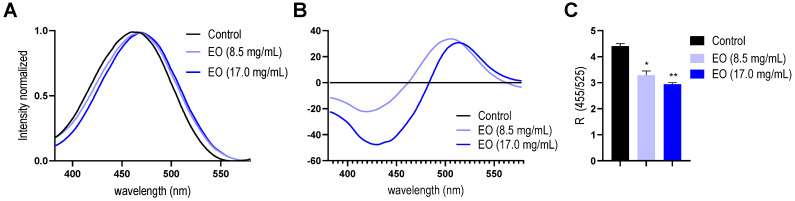
Effect of EO of *Tagetes minuta* on membrane dipole potential measured by Di-8-ANEPPS fluorescence spectroscopy on liposomes. (**A**) Excitation spectra of liposome suspensions loaded with Di-8-ANEPPS incubated at different EO concentrations. (**B**) Differential fluorescence spectra of Di-8-ANEPPS-loaded liposomes after EO incubation. (**C**) Changes in the magnitude of the dipole potential, expressed as the fluorescence ratio R (455/525). The data presented are an average of three separate measurements, and the error bars show the standard deviation of the averaged results. **, *p* < 0.05. *, *p* < 0.01. One-way ANOVA followed by Dunnett’s test for multiple comparisons vs. control.

First, the changes induced by the EO on the electrical property dipole potential were assessed by Di-8-ANEPPS fluorescence spectroscopy. If a molecule interacts with the membrane by entering or adsorbing, it is expected that the membrane order changes to some extent, resulting in fluctuations in its dipole potential. These alterations can be recorded by shifts on the excitation spectrum of Di-8-ANEPPS [39,40]. It was observed that the EO led a spectral shift to higher wavelengths, from 461 nm to 469 nm, on the excitation spectra of Di-8-ANEPPS on liposomes (Figure 4A). This effect can be more easily seen in the differential spectra obtained by subtracting the spectra of lipid membranes with and without EO incubation (Figure 4B). This behavior was dose-dependent, indicating there are significant changes in the membrane dipole potential at concentrations of EO where the antibacterial activity was observed. In order to quantify the EO effect on this electrical property, the R values were obtained from the ratio between the fluorescence intensities at 455 nm and 525 nm. The R-value decreases significantly as the EO concentration increases (Figure 4C). These results confirm the ability of some components of the EO to interact with the lipid membrane. It was previously pointed out that many phytochemicals, such as flavonoids, alkaloids, terpenoids, stilbenoids, and related molecules, which are relatively lipophilic, may interact with the lipid membrane, altering the properties of the lipid matrix, specifically by affecting the transmembrane distribution of the electrical potential [41]. In this way, the reduction in the dipole potential observed in this study could be explained by the electrical interaction of the molecule dipole moment of certain polar EO molecules, such as (*E*)-Tagetone, (*Z*)-Ocimenone, and Thymol, into the lipid bilayer, which is opposite to the membrane dipole moment of the phospholipid chemical groups. 

The rearrangements of phospholipids in the membrane caused by the partition between them and the aqueous medium of EO more lipophilic molecules could also lead to alterations in the transition temperature (Tm) of the membrane. It should be pointed out that all majority compounds of the EO, especially Pinene and Limonene, showed high octanol/water partition coefficients, which allowed them to partition in the lipid phase. For that reason, the Tm of liposomes with the same composition (DMPC: DMPG 5:1) incubated with and without EO was determined by DLS measurements. This approach assesses changes in lipid optical characteristics by varying the average count rate. Thus, plotting the mean count rates as a function of temperature allows one to determine the Tm and cooperativity of the process [42,43].

**Figure 5:**
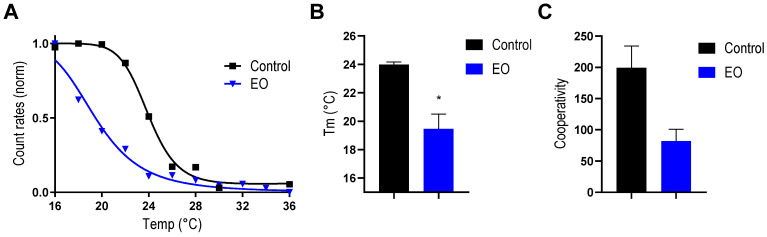
Effect of the *Tagetes minuta* EO on membrane transition temperature (Tm) of the lipid membrane: (**A**) Count rates as a function of temperature; (**B**) transition temperature; and (**C**) degree of cooperativity of phospholipid in membrane transition. The data presented are an average of three separate measurements, and the error bars show the standard deviation of the averaged results. *, *p* < 0.01. Unpaired *t*-test.

As can be seen in Figure 5, the incubation of liposomes with EO produces notable changes in the count rate behavior that result in a significant reduction in the Tm (≅4 °C) and in the cooperativity of this transition.

Previously, it was reported that changes in Tm can be used as an indication of the interaction of exogenous substances with the acyl chains of lipids; therefore, these variations could be ascribed to the partition of some EO components in the hydrophobic core of the lipid membrane [44]. In this context, the obtained results reinforce those obtained by Di-8ANNPES, confirming the interaction of some components of the EO with the lipid bilayer. In the same line, the reduction in the cooperativity on the lipid phase transition also confirms the partition of compounds into the lipid bilayer, as cooperativity rates the sharpness of the phase transition and is highly dependent on the presence of any additive in the lipid bilayer [45]. 

**Figure 6:**
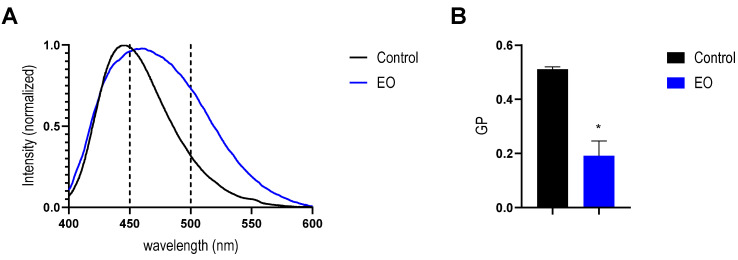
Effect on membrane polarity of *Tagetes minuta* EO in lipid vesicles. (**A**) Laurdan emission spectra on liposomes incubated with and without EO. Dashed lines correspond to wavelengths used for GP calculation (**B**). GP values of liposomes with and without EO. The data presented are an average of three separate measurements, and the error bars show the standard deviation of the averaged results. *, *p* < 0.01. Unpaired *t*-test.

Finally, to complement the results presented above, changes in membrane order were evaluated by using Laurdan. The interaction of the *T. minuta* EO with the biomimetic bacterial membranes produces a significant red shift in Laurdan emission spectra (Figure 6A). From the experimental data, the GP values were calculated. It was observed that GP value significantly decreased from 0.51 ± 0.01 for control liposomes to 0.19 ± 0.07 for liposomes incubated with *T. minuta* EO (Figure 6B). This result indicates a change in membrane polarity that could be explained by the increase in water molecules into the membrane. It is worth noting that the GP values of control liposomes agree with the values previously published [36,46]. A lower GP value is associated with an increase in membrane fluidity [47]. Furthermore, this dramatic change in the GP value is consistent with a phase transition of the lipid bilayer from a gel to a liquid crystalline state [47], driven by the interaction of EO components with the membrane. Taking into account that this experiment was carried out at 21 °C, the phase transition observed is consistent with the changes in the Tm obtained by DLS (Figure 5). 

The strength of van der Waals interactions among adjacent lipids determines the physical state of the lipid bilayer [42]. Therefore, these results obtained by Laurdan and DLS could be explained by structural changes at the membrane level (like bending, lateral diffusion, expansibility, bending, and permeability) induced by the partition of different compounds of the EO throughout the thickness of the membrane. The insertion of these compounds into the membrane could contribute to the energy required to disturb the lipid contacts, which results in a more fluid and labile membrane. 

**Figure 7:**
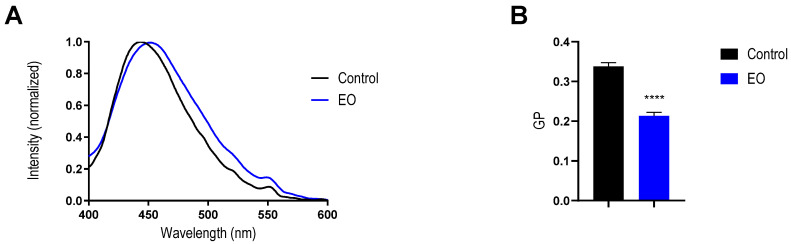
Effect on membrane polarity of the *Tagetes minuta* EO on *Staphylococcus aureus* membrane. (**A**) Laurdan emission spectra on *Sthapylococcus aureus* suspensions incubated with and without EO (**B**). GP values of *Staphylococcus aureus* with and without EO. The data represent an average of three independent measurements; the error bars indicate the standard deviation of the averaged values. ****, *p* < 0.0001. Unpaired *t*-test.

Overall, all these data from model membranes confirm that certain components of the EO can induce several changes in the membrane features, including a reduction in the dipole potential and significant changes in the Tm of the lipid bilayer. This membrane rearrangement induced by the EO components could explain the rise in the membrane permeability found in both bacteria used. This kind of membrane perturbation was previously reported for other EOs, as well as different pure monoterpenes [36,44,45,48], and cyclic compounds that might diffuse to the membrane and increase its fluidity [49]. To confirm the impact of EO on the bacterial membrane phase state, we also evaluated the effect on membrane fluidity by using Laurdan directly on *S. aureus* cells (Figure 7). The GP value found for control *S. aureus* (0.34 ± 0.01) matches with previous reports in the literature [50,51]. After the incubation with the EO at MIC, the GP value was reduced to 0.21 ± 0.01, in good agreement with the data obtained using liposomes. This result confirms that the effects described previously for model membranes also take place on the bacterial membrane of *S. aureus*. Therefore, we can prove that the EO was able to perturb the bacterial membrane packing, resulting in an increase in its permeability, which led to bacterial death. 

## 3. Conclusions

In summary, in this work, we found that the EO obtained from *T. minuta* from Santiago del Estero (Northwest Argentina) showed antibacterial activity against Gram-negative *E. coli* and Gram-positive *S. aureus*. Furthermore, this activity demonstrated a high level of lethality against both bacteria in a short time of incubation (1 h), where in the case of *E. coli*, all bacteria were killed in this period. 

In addition, for the first time, we investigated the mechanism of action of this EO, showing that the bacterial membrane is one of its targets. The obtained results allow us to conclude that some oil components can be inserted into the lipid bilayer and induce some membrane structure rearrangements, resulting in a more fluid and permeable membrane, compromising its barrier effect and causing bacterial mortality. It was previously pointed out for other EOs that the bacterial membrane is the target for the antimicrobial activity of EOs due to the hydrophobic nature of EO components [52].

Knowing that understanding the mechanism of action is a critical step for future applications of novel natural antimicrobials, the results obtained in this work represent an important milestone for unraveling the full potential of the *T. minuta* EO. 

## 4. Materials and Methods

### 4.1. Materials

DMPC (1,2-dimyristoyl-sn-glycero-3-phosphocholine) and DMPG (1,2-dimyristoyl-sn-glycero-3-phospho-1′-rac-glycerol) were obtained from Avanti Polar Lipids (USA). Sodium sulfate anhydride was purchased from Sigma-Aldrich (St. Louis, MO, USA). The water used was ultra-pure (conductivity: 0.002 ± 0.001 μS/cm; pH = 5.0). The organic solvents used (chloroform, methanol, hexane, ethyl acetate, and DMSO) were all analytical grade, purchased from Merck Química SAIC (Argentina). For the microbiological tests, Mueller Hinton broth (MH broth) or Muller Hinton Agar (MH Agar), obtained from Britania (Buenos Aires, Argentina), was used. 

### 4.2. Bacterial Strains 

The reference strains *S. aureus* ATCC 25,923 and *E. coli* ATCC 25,922 were used. The stocks of each bacterium were conserved on glycerol (20% *v*/*v*) at −20 °C. To carry out all the assays, strains were grown in MH broth at 37 °C. 

### 4.3. Plant Material, Isolation, and Characterization of Essential Oil

Samples of *T. minuta* were collected in 2023 at La Banda, Santiago del Estero, Argentina (27°43′05.6″ S 64°17′13.7″ W). The plant was identified, and the voucher specimen was deposited at the herbarium of the Cátedra de Botánica Agrícola of the Facultad de Agronomía y Agroindustrias, Universidad Nacional de Santiago del Estero (accession number: EP N°453). The aerial parts of *T. minuta* (ca. 200 g) were hydro-distilled for 4 h using a Clevenger-type apparatus. Volatile oils were analyzed using GC-FID and GC-MS chromatography as described in [53]. The percentages represent the average value of three separate analyses. The chemicals were identified by calculating retention indices and comparing them to commercial standards and literature data. The collected mass spectra were analyzed using the NIST 08 database [54]. The obtained EO of *T. minuta* was solubilized in DMSO for biological assays; therefore, control experiments with DMSO were conducted to match the solvent concentrations present in the EO solutions tested. In all the cases at the maximum concentration of EO used, the final DMSO concentration was less than 2% *v*/*v*. 

### 4.4. MIC and MBC Determinations

The current study employed the Minimum Inhibitory Concentration (MIC) and Minimum Bactericide Concentration (MBC) to obtain the lowest concentration of EO with no visible bacterial growth and the lowest concentration that completely eliminates the growth on agar plates [55]. The MIC assays were performed according to the Clinical and Laboratory Standards Institute guidelines [56]. First, the EO stock in DMSO was inoculated in a 96-well flat-bottom polystyrene plate, obtaining different concentrations between 17 mg/mL and 0.016 mg/mL. Subsequently, 100 µL microbial suspensions (1 × 10^6^ cfu/mL) were added, followed by incubation at 37 °C for 24 h. Concerning the MBC, 10 μL were taken from the wells without visual bacteria growth and transferred on Petri dishes with an MH agar medium and incubated at 37 °C for 24 h [36]. 

### 4.5. Bacterial Viability 

For a better characterization, the viable bacteria after 1 h incubation with the EO, at MIC, was obtained by counting colony-forming units (CFUs). The same conditions were followed with the addition of DMSO or gentamicin (at MIC) as control. Following incubation, a serial dilution by a factor of ten was obtained and plated in MH agar media. Finally, plates were incubated at 37 °C for 24 h. The colonies were counted, and viable bacteria were expressed as CFU/mL using the following Equation (1): N_f_/N_o_ = (∑C_f_/V × D)/(∑C_0_/V × D), where N_f_ and N_o_ are the final and initial counts of CFU, respectively; ∑C is the average of the colonies counted on the plates, which should contain between 30 and 300 colonies; V is the volume of inoculum used in each plate, in milliliters; and d is the corresponding dilution.

### 4.6. Fluorescence Microscopy

To evaluate the bacterial membrane integrity, SYTO-9 and propidium iodide (PI) were used to observe the bacterium, which was incubated with the EO for 1 h. This protocol was carried out following the procedure described by Cutro et al. [36]. In brief, each strain’s overnight culture was inoculated into MH broth and incubated at 37 °C with agitation until an OD 600 of 0.3 was reached. Afterward, the bacterial suspensions were incubated with the EO at MIC for 24 h. For the control, the same conditions were followed with the addition of DMSO. Ending the incubation period, each bacterial suspension was incubated with SYTO-9 and PI at a final concentration of 6 µM and 30 µM, respectively, for 5 min at 37 °C under agitation and protected from the light. Finally, the samples were observed using an inverted fluorescence microscope (Olympus CKX 41, Tokyo, Japan) coupled with an Olympus QColor3-RTV-R digital camera.

### 4.7. Fluorescence Spectroscopy

The proportion of membrane-damaged bacteria was also determined using fluorescence spectroscopy following manufacturing instructions (BacLight bacterial viability kit L7007, Molecular Probes, Thermo Fisher Scientific, Waltham, MA, USA), with modifications. Briefly, each strain’s overnight culture was inoculated into MH broth and incubated at 37 °C with agitation until an OD 600 of 0.6 was reached. Then, the bacteria suspensions were washed three times in PBS (10 mM, pH = 7.40). Bacterial suspensions were fractionated and incubated with different treatments: (a) antimicrobial agent: bacterium suspension + EO at MIC; (b) viable bacteria control: bacterium suspension + DMSO in the highest amount; (c) killed bacteria control: bacterium suspension + 70% isopropyl alcohol (for killing bacteria). After incubation, all the treatments were washed two times, and the OD 600 was adjusted to 0.3. Afterward, each treatment was incubated with SYTO-9 and PI at a final concentration of 33.4 µM and 200 µM, respectively, for 15 min at 37 °C under agitation and protected from the light. Finally, fluorescence emission spectra were registered in a fluorescence spectrometer Cary Eclipse (Agilent, USA), with excitation at 480 nm and emission from 500 to 700 nm. From the spectroscopic data, the green/red fluorescence ratio was calculated by using the following Equation (2): R = I_510/_I_620_, where I_510_ is the fluorescent intensity at 510 nm, and I_620_ is the fluorescent intensity at 620 nm.

### 4.8. Zeta Potential

The Zeta potential was employed to determine the interaction of EO with the bacterial surface. This protocol was carried out following the procedure described by Cutro et al. [33]. Briefly, each strain’s overnight culture was inoculated into MH broth and incubated at 37 °C with agitation until an OD 600 of 0.15 was reached. Then, aliquots of bacteria suspensions were incubated with the EO at 0.5 × MIC and MIC for 1 h. As a control, the same conditions were followed with the addition of DMSO instead of EO. Each suspension was centrifuged for 5 min at 5000 rpm, and the obtained pellets were suspended in PBS (20 mM, pH = 7.40). This procedure was repeated twice, and the washed suspensions were employed for Zeta potential determination using a Horiba SZ-100 nanoparticle analyzer equipment (Horiba, Kyoto, Japan). The applied voltage and filters were automatically adjusted, maintaining a constant temperature of 25 °C. The Zeta potential was calculated from cell mobility by using the Smoluchowski model (Equation (3)): Zeta potential = (μE η)/ε *f (kr)*, where μE is the electrophoretic mobility, η is the dispersion medium viscosity, ε is the dispersion medium dielectric constant, and f is Henry’s function [29,33]. 

### 4.9. Liposome Preparations 

Many researchers have utilized lipid membranes as biological membrane models. The benefits of this approach are system simplicity, the possibility of modulating their lipid composition to mimic different types of membranes, and studying interactions with antimicrobial agents [57]. In this study, multilamellar vesicles (MLVs) of a mixture of DMPC/DMPGs (5:1) were employed as a model of bacterial membrane. To obtain multilamellar MLVs, stock solutions of each lipid (10 mM) were prepared by dissolving the respective solid lipid in a chloroform and methanol (5:1 *v*/*v*) mixture. Subsequently, by gently evaporating the solvent under a nitrogen stream, a lipid film was obtained. To ensure that all the solvent had evaporated, the film was subjected to a vacuum. The dried film was hydrated in PBS (20 mM, pH = 7.40) at 40 °C for 60 min. Finally, the MLVs obtained were extruded (pore diameter = 100 nm, Avanti Polar Lipids, USA), according to the procedure of Szoka (1980) [58], to obtain large unilamellar vesicles (LUVs).

### 4.10. Dipole Potential

The LUV suspension (500 μM of phospholipids) was incubated with a di-8-ANEPPS probe at a 10 μM final concentration for 24 h at 37 °C with continuous shaking and protected from the light. On the other hand, an identical suspension of LUV (500 μM) without a probe was reserved to be used as a dispersion measurement control. Then, the suspension was fractionated and incubated for 1 h with EO (17.0 mg/mL) and DMSO (8.8 μL/mL) at 37 °C with continuous shaking and protection from the light. Finally, fluorescence excitation spectra of the samples were recorded in the fluorescence spectrometer Cary Eclipse in a range of 380 to 580 nm, setting the emission at 611 nm. From the spectroscopic data, the magnitude of the dipole potential change (R) was calculated by using the following equation adapted from Maturana et al. [59]: R = I_455/_I_525_, where I_455_ is the fluorescent intensity at 455 nm, and I_525_ is the fluorescent intensity at 525 nm. 

### 4.11. Transition Temperature (Tm) by DLS 

The Tm of LUVs (850 µM) incubated with 17.0 mg/mL of EO was evaluated by dynamic light scattering. Count rates as a function of temperature were recorded in a Horiba SZ-100 nanoparticle analyzer equipment. From the data obtained, the Tm and cooperativity were obtained by using the following Equation (4): Meant count rare = r_s1_ + p_1T_ + (r_s2_ − r_s1_ + p_2T_ − p_1T_)/(1 + 10 ^ (B (1/T − 1/Tm))), where T is the temperature (°C), p1 and p2 represent the slopes of the straight lines at the beginning and the end of the plot, and rs1 and rs2 are the count rate intercepting values at the y-axis [60]. Finally, the cooperativity (B) and Tm were obtained by fitted data [60]. As a control, the highest amount of DMSO tested was used. 

### 4.12. Laurdan Fluorescence Spectroscopy in LUVs

To obtain Laurdan-loaded liposomes, aliquots of Laurdan from a stock solution in methanol were added to lipids dissolved in chloroform/methanol (2:1) to achieve a 1:300 (Laurdan/lipid) ratio. Then, the LUVs suspensions were prepared as detailed above. Afterwards, the LUV suspension (400 μM of phospholipids) obtained was fractionated and incubated for 1 h with EO (17.0 mg/mL) or DMSO, respectively. After that, Laurdan fluorescence emission was obtained with a Cary Eclipse spectrofluorometer. The temperature was set at 21 °C and controlled by a circulating water bath. The Laurdan emission spectra were recorded from 400 nm to 600 nm, using a 350 nm excitation wavelength. A blank spectrum was obtained with unlabeled liposomes and was recorded. At the concentrations of LUV and bandpass used, the interference from dispersed light was negligible. From the obtained data, the Laurdan Generalized Polarization (GP) was determined by using the following equation adapted from Parasassi et al. [61]: GP = (I_450_ − I_500_)/(I_450_ + I_500_), where I_450_ is the fluorescent intensity at 450 nm, and I_500_ is the fluorescent intensity at 500 nm.

### 4.13. Laurdan Fluorescence Spectroscopy in Bacteria

An overnight culture of *S. aureus* was inoculated in MH broth and incubated under agitation at 37 °C until reaching an OD 600 of 0.5. Then, bacteria suspensions were incubated with the EO at MIC for 1 h. For the control, the same conditions were followed with the addition of DMSO. On the other hand, a remaining aliquot of bacterium suspension was reserved to be used as a dispersion measurement control. After incubation, all the treatments were washed two times with PBS (10 mM, pH = 7.40), and the OD600 was adjusted to 0.2. After that, the suspensions were incubated with a Laurdan probe at 8 μM final concentration for 30 min at 37 °C with continuous shaking and protected from the light. Then, the suspensions were washed once again with PBS (10 mM, pH = 7.40). Finally, fluorescence emission spectra of the samples were recorded in the fluorescence spectrometer CARY Eclipse, in a range of 400 to 600 nm, setting the emission at 350 nm. From the obtained data, the GP was calculated by using the equation adapted from Bessa et al. [62] as follows: GP = (I_440_ − I_490_)/(I_440_ + I_490_), where I_440_ is the fluorescent intensity at 440 nm, and I_490_ is the fluorescent intensity at 490 nm.

## Figures and Tables

**Table 1:** Chemical composition of the essential oil of *Tagetes minuta*.

Peak	Compound ^1^	Area (%)	RI ^2^
1	β-Pinene	12.34	1004
2	Limonene	7.93	1024
3	α-Pinene epoxide	1.19	1045
4	β-Ocimene	0.35	1054
5	Dihydrotagetone	1.25	1063
6	γ-Terpinene	0.85	1078
7	1-Octanol	0.49	1086
8	α-Terpinolene	10.75	1099
9	(*E*)-Tagetone	16.60	1129
10	(*Z*)-Tagetone	0.84	1153
11	Terpinen-4-ol	0.73	1170
12	Carvyl acetate	0.86	1186
13	Verbenone	0.41	1192
14	Thymol	6.12	1210
15	(*Z*)-Ocimenone	12.57	1219
16	(*E*)-Ocimenone	4.92	1257
17	Bornyl acetate	2.27	1300
18	Isolongifolene	0.94	1332
19	β-Resorcylaldehyde	0.54	1346
20	β-Elemene	1.27	1356
21	(*Z*)-Jasmone	5.61	1378
22	Isocaryophillene	2.66	1395
23	(*E*)-Caryophyllene	2.12	1434
24	α-Cadinene	0.62	1446
25	α-Humulene	1.20	1459
26	Bicyclogermacrene	1.74	1473
27	Spathulenol	2.83	1563
-	**Total**	100	-

^1^ Compound identification based on RI, NIST 08 library, and bibliography. ^2^ RI, experimental linear retention indices on TR5-ms column.

**Table 2:** Minimum inhibitory concentration (MIC) and minimum bactericidal concentration (MBC) against *Staphylococcus aureus* and *Escherichia coli* from *Tagetes minuta* EO.

*Staphylococcus aureus*		*Escherichia coli*	
MIC (mg/mL)	MBC (mg/mL)	MIC (mg/mL)	MBC (mg/mL)
8.5	>17.0	17.0	17.0

## Data Availability

The raw data supporting the conclusions of this article will be made available by the authors on request.

## Supplementary Materials

The following supporting information can be downloaded at https://www.mdpi.com/article/10.3390/antibiotics14070632/s1: Figure S1: Relative membrane damage of *E. coli* and *S. aureus* stained using the probes SYTO 9 and PI after 1 h of incubation with EO at MIC.

## Abbreviations

The following abbreviations are used in this manuscript:

DMPC	1,2-dimyristoyl-sn-glycero-3-phosphocholine
DMPG	1,2-dimyristoyl-sn-glycero-3-phospho-1′-rac-glycerol
DMSO	Dimethyl sulfoxide
EO	Essential oil
GP	Generalized Polarization
LUV	Large unilamellar vesicles
MBC	Minimal bactericidal concentration
MH	Muller Hinton
MIC	Minimal inhibitory concentration
MLV	multilamellar vesicles
PI	Propidium iodide
RI	Retention index
