# Supplementary material for: Antimicrobial Action of Essential Oil of Tagetes minuta: Role of the Bacterial Membrane in the Mechanism of Action
Source: Antibiotics (Basel). 2025 Jun 21;14(7):632. doi: 10.3390/antibiotics14070632 (PMC12291637; doi:10.3390/antibiotics14070632)

Supplementary Material

Antimicrobial Action of Essential Oil of *Tagetes minuta* Role of the Bacterial Membrane in the Mechanism of Action

Anahí Bordón [1], Sergio A. Rodríguez [2,*], Douglas Siqueira de Almeida Chaves [3], Andrea C. Cutró [1,4,*] and Axel Hollmann [1,5,*]

[1] Laboratorio de Compuestos Bioactivos, Centro de Investigación en Biofísica Aplicada y Alimentos (CIBAAL), Universidad Nacional de Santiago del Estero, Consejo Nacional de Investigaciones Científicas y Técnicas (CONICET), RN 9 km 1125, Santiago del Estero 4206, Argentina; ani.bordon.1996@gmail.com

[2] Instituto de Ciencias Químicas, Facultad de Agronomía y Agroindustrias, Universidad Nacional de Santiago del Estero, CONICET, RN 9 km 1125, Santiago del Estero 4206, Argentina

[3] Pharmaceutical Science Department, Health and Biological Science Institute, Federal Rural University of Rio de Janeiro, BR 465, km 7, Seropédica 23897-000, Rio de Janeiro, Brazil; chavesdsa@ufrrj.br

[4] Facultad de Ciencias Médicas, Universidad Nacional de Santiago del Estero, Calle Reforma del 18 N° 1234, Santiago del Estero 4200, Argentina

[5] Laboratorio de Microbiología Molecular, Universidad Nacional de Quilmes. Roque Sáenz Peña 352, Bernal 1876, Argentina

* Correspondence: drsergiorod@gmail.com (S.A.R.); cutro.andrea@gmail.com (A.C.C.); ahollmann@conicet.gov.ar (A.H.)

Figure S1. Relative membrane damage of *Staphylococcus aureus* and *Escherichia coli* stained using the probes SYTO 9 and PI after 1 h of incubation with EO at MIC concentration.

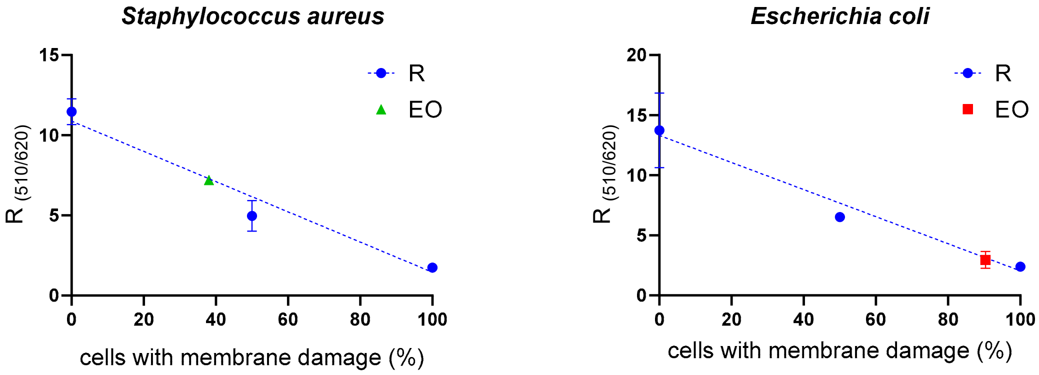

Supplement: Supplementary file 1 [file antibiotics-14-00632-s001.zip › antibiotics-3703355-supplementary.pdf]
